# Differential expression of microRNAs in preneoplastic gastric mucosa

**DOI:** 10.1038/srep08270

**Published:** 2015-02-05

**Authors:** Alexander Link, Wiebke Schirrmeister, Cosima Langner, Mariya Varbanova, Jan Bornschein, Thomas Wex, Peter Malfertheiner

**Affiliations:** 1 Department of Gastroenterology, Hepatology and Infectious Diseases, Otto-von-Guericke University, Magdeburg, Germany

## Abstract

Gastric carcinogenesis is a multifactorial *H.pylori*-triggered dynamic process that goes through a cascade of preneoplastic conditions. The expression of miRNAs in the stomach with regard to preneoplastic precursor conditions and *H.pylori* infection has not been investigated systematically. In this prospective proof-of-principle study, we evaluated the miRNA expression in gastric antrum and corpus mucosa from patients with chronic non-atrophic gastritis (CNAG), atrophic gastritis (AG), and GC compared to controls. Gastric normal mucosa shows a unique expression pattern for miR-21, miR-155 and miR-223, which is specific for different regions. In correlation with progression of Correa's cascade and *H.pylori* infection, we observed a gradual increase in miR-155 and miR-223 both in corpus and antrum and miR-21 only in the antrum mucosa. Using miRNA expression we calculated a score that allowed us to discriminate patients with AG from subjects with normal mucosa with high diagnostic accuracy in testing and validation cohorts reproducibly. In summary, the expression pattern of miRNAs in the gastric mucosa is gradually increased with progression of Correa's cascade and *H.pylori* infection, suggesting miRNAs as potential biomarkers for preneoplastic precursor conditions. However, differences of miRNA expression between the gastric antrum and the corpus need to be considered in future studies.

Gastric cancer (GC) remains one of the leading health burdens accounting for up to 8.8% of all cancers and is the third leading cause of cancer-related deaths worldwide^1^. GC is a consequence of multiple factors including genetic predisposition, environmental factors, diet and aging. Among these, chronic inflammation due to *H.pylori* infection plays the key role in triggering carcinogenesis. According to a multistep process first described by Correa, development of intestinal type gastric cancer is triggered by *H.pylori* driven inflammation leading to typical stages of mucosal alterations such as chronic gastritis, glandular atrophy, intestinal metaplasia, dysplasia before reaching the final stage of invasive GC^2^. Even though the relative number of individuals that progress from preneoplastic stages such as atrophic gastritis (AG) to GC might be low, the absolute individual risk for gastric malignancy increases substantially with various factors including host genetic predisposition, hereditary and acquired epigenetic factors, and specific *H.pylori* strains with more pathogenic bacterial virulence factors (cag-PAI, vacA, etc.)^3^,^4^. Although a number of potential risk factors have been widely studied, currently only histological evaluation of the mucosa has been applied more or less successfully in clinical routine for an individual gastric cancer risk stratification^5^. Current recommendation in the management of patients with high risk gastritis include endoscopic surveillance with histological staging of the gastric mucosa according to the updated Sydney classification or the Operative Link on Gastritis Assessment (OLGA) and Operative Link on Gastric Intestinal Metaplasia Assessment (OLGIM) staging systems^6^. However, only a minority of high risk patients will develop GC. Therefore, in order to avoid unnecessary interventions, there is a need for suitable molecular biomarkers that may predict more precisely the risk for gastric cancer development both in low- and high-risk populations.

Noncoding microRNAs (miRNAs) are widely established as an outstanding class of RNA molecules that has unique features suitable for biomarker use. MiRNAs display an exceptional stability against degradation and are easily extracted from various specimens including tissues, blood, feces etc.^7^,^8^. Differential expression of miRNAs has been reported for various tumor entities including gastric cancer^7^,^9^,^10^. Especially increased expression of miR-21 is identified both in tumor tissues and in blood of GC patients when compared to controls^11^ and its oncogenic role has been widely studied^10^,^12^,^13^,^14^. However, the understanding of miRNA deregulation in the stomach related to *H.pylori* infection and different stages of progression according to Correa's cascade is still preliminary^7^. In one of the earliest studies, Petrocca et al. showed that chronic inflammation of the gastric mucosa was associated with alteration of seven miRNAs, specifically miR-155^15^. In vitro functional analysis revealed an essential role of miR-155 in modulation of *H.pylori*-triggered mucosal inflammation^16^,^17^. Furthermore, Matsushima et al. profiled tissue samples from *H.pylori* positive and -negative subjects and described a downregulation of 30 miRNAs and an upregulation of miR-223^18^. *H.pylori* eradication was associated with at least partial reversal of alterations of miRNA expression already 4 weeks after successful therapy^18^. Plasma analyses revealed also increased miR-223 levels in *H.pylori* positive subjects^19^. Nevertheless, these miRNAs are not sufficiently studied in systematic manner in *H.pylori*-associated chronic gastritis or preneoplastic gastric conditions such as AG with and without intestinal metaplasia (IM).

In this study, we aimed first to characterize the expression of miRNA in the gastric mucosa in relation to preneoplastic mucosal alterations and to anatomical sites of the stomach, and second to evaluate the diagnostic potential of the investigated miRNAs as biomarker for AG (Suppl. Figure S1). Using a subset of miRNAs that are frequently associated with GC (miR-21, miR-155, miR-223), we show that the gastric corpus and the antrum exhibit different miRNA expression levels. Additionally, we show that these miRNAs are differentially expressed in the mucosa of patients with chronic non-atrophic gastritis (CNAG), AG and GC, suggesting their potential use as biomarkers for gastric preneoplastic conditions.

## Results

### Differential miRNA expression in gastric cancer compared to normal gastric tissue

To investigate the miRNA expression in the gastric mucosa systematically, we selected miR-21, miR-155 and miR-223. Expression of miR-21 is frequently deregulated in tumor samples, specifically in gastric neoplasia^7^. MiR-155 is involved in the T-cell dependent immunological response related to *H.pylori* infection^17^. MiR-223 is a gastritis-associated marker for *H.pylori* infection which is highly expressed in neutrophil granulocytes^20^. There is an increased expression of miR-21 and miR-223 in GC tissues compared to antrum and corpus mucosa samples from control subjects with normal gastric mucosa (N) (1.25 ± 1.06 vs. 0.26 ± 0.08, p<0.0001; 0.05 ± 0.05 vs. 0.56 ± 0.95, p < 0.001, respectively) (Suppl. Figure S2). The difference in the expression of miR-155 in biopsies from GC compared to controls did not reach statistical significance (N: 0.01 ± 0.01 vs. GC: 0.03 ± 0.03, p = 0.46).

### Differences in miRNA expression between corpus and antrum

**Figure 1:**
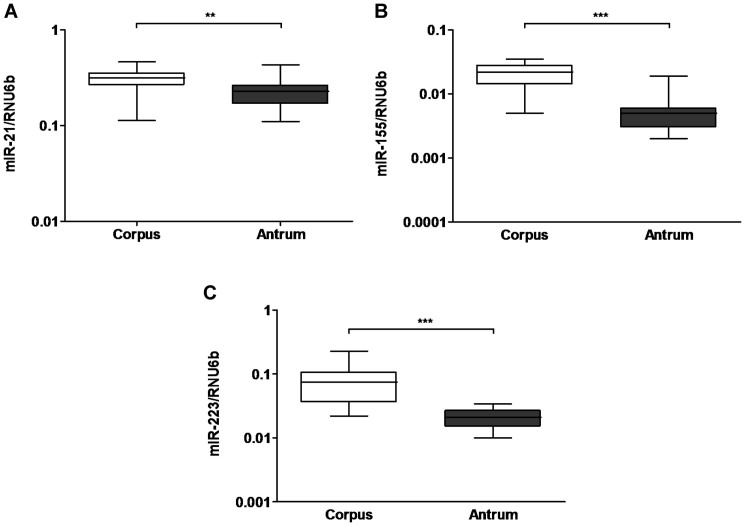
Regional differences in miRNA expression in the stomach mucosa. Expression of miR-21 (A), miR-155 (B) and miR-223 (C) were evaluated in paired corpus and antrum samples of the stomach (each n = 19). The values represent 2^ΔdCt^-values normalized to RNU6b. ***-p < 0.0001; scatter-plot, horizontal line represent medians.

Gastric biopsies or surgical specimens are frequently used for molecular analyses, however, the potential difference between the anatomical regions of the stomach has not been evaluated for miRNA expression yet. Therefore, we first compared the expression of miR-21, miR-155 and miR-223 in corpus and antrum samples from controls normal *H.pylori*-negative mucosa. All three studied miRNAs showed significantly lower expression in the antrum compared to the corpus mucosa in paired analyses. As shown in the Figure 1, the difference was more pronounced for miR-155 (corpus 0.02 ± 0.009 vs. antrum 0.006 ± 0.004) and for miR-223 (0.078 ± 0.05 vs. 0.02 ± 0.007) than for miR-21 (0.3 ± 0.08 vs. 0.22 ± 0.07). Similar expression patterns were observed in the mucosa of patients with chronic gastritis although the difference in the expression of miR-21 did not reach statistical significance (data not shown). Therefore, for further analyses, the miRNA expression was evaluated separately for different regions.

### Alterations in the miRNA expression in correlation with different stages of Correa's cascade

**Figure 2:**
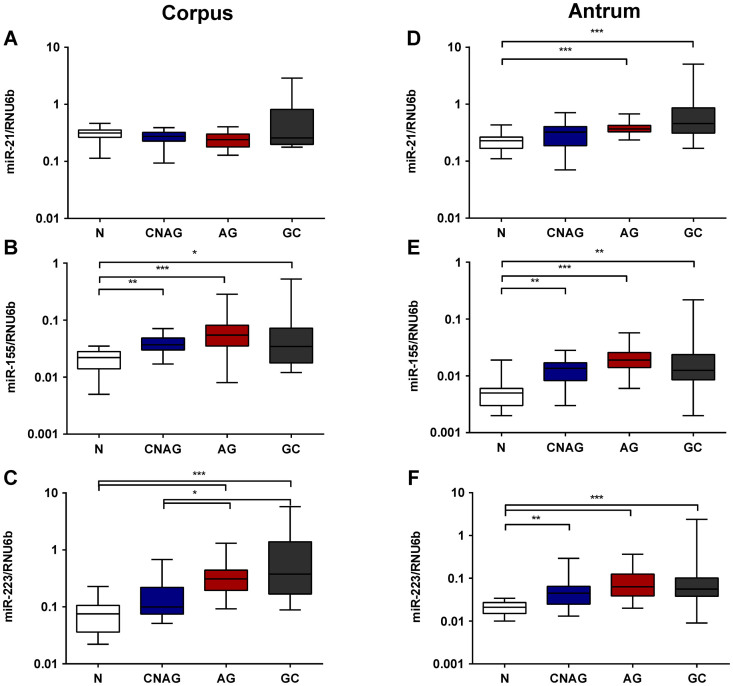
miRNA expression alterations in chronic gastritis and preneoplastic mucosal conditions. The expression of miR-21, miR-155 and miR-223 is shown as 2^ΔdCt^-values normalized to RNU6b for corpus (A–C) and antrum (D–F), respectively. N – controls (n = 19); CNAG – chronic non-atrophic gastritis (n = 25); AG – atrophic gastritis (n = 20); GC- tumor free antrum (n = 16) or corpus (n = 14) tissue from patients with gastric cancer. ***-p < 0.0001; **-p < 0.001; *- p < 0.05.

There are only few studies available that evaluate the association of miRNA expression with mucosal changes in subjects with chronic gastritis. We looked at miRNA expression in relation to various pathological conditions of the gastric mucosa (including GC) in a well characterized prospectively collected cohort of samples (Table 1). As demonstrated in Figure 2, we did not find a significant difference in miR-21 expression in the corpus mucosa among the groups. The expression of miR-155 and miR-223 showed a stepwise increase from normal *H.pylori*-negative gastric mucosa to CNAG, AG and corpus mucosa from patients with GC (Figure 2A-C). The biggest increase in expression was observed in mucosa with AG while only non-significant changes were found between AG and corpus mucosa from GC patients. Similarly, expression of miR-21, miR-155 and miR-223 showed an increase from normal to CNAG, AG and GC antrum mucosa (Figure 2D–F). With respect to regional differences in the miRNA expression, miRNA expression in both corpus and antrum did not differ significantly between patients with preneoplastic conditions and mucosa from GC patients (Figure 2).

### H.pylori-related alterations in miRNA expression

**Figure 3:**
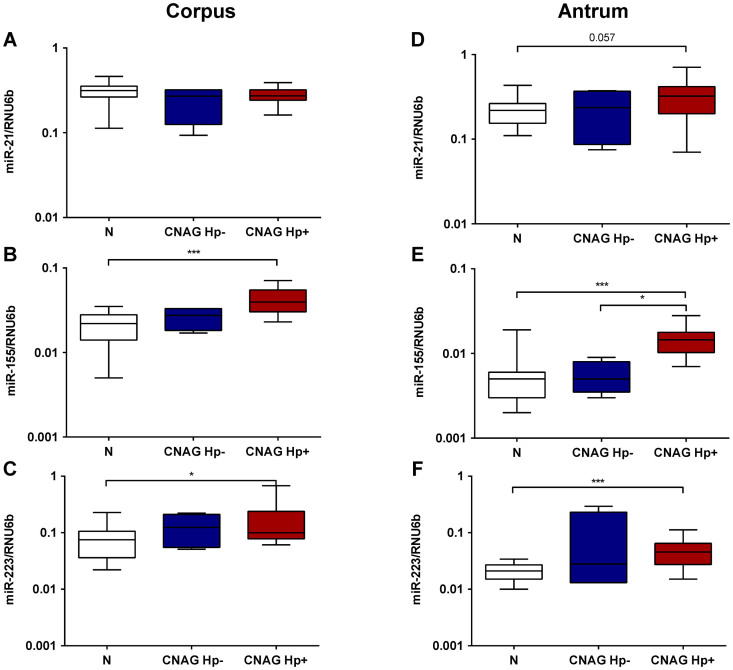
*H.pylori* infection and changes in miRNA expression in chronic gastritis patients. The expression of miR-21, miR-155 and miR-223 was evaluated in controls (N; n = 19), patients with chronic non-atrophic gastritis without evidence or present or past *H.pylori* infection (CNAG Hp-; n = 4) and patients with *H.pylori* positive gastritis (CNAG Hp+; n = 21). The values are presented as 2^ΔdCt^-values normalized to RNU6b for corpus (A–C) and antrum (D–F) separately. ***-p < 0.0001; **-p < 0.001; *- p < 0.05.

*H.pylori* infection has been associated with alterations in the miRNA expression pattern^21^. Having shown a stepwise increase of the miRNA expression according to the stages of Correa's cascade, we questioned if these alterations might be related to *H.pylori* infection. Indeed, miR-155 and miR-223, both in corpus and antrum, were strongly elevated in patients with *H.pylori*-positive CNAG compared to controls (Figure 3). Although the miRNA expression was also slightly increased in the mucosa of *H.pylori*-negative CNAG the differences did not reach statistical significance. MiR-21 expression analyses revealed only a non-significant trend for increased expression in the antrum (p = 0.057) and no difference for the corpus (Figure 3A and D). Subgroupanalyses of *H.pylori* infection in patients with AG revealed only insignificant differences of miRNA expression between patients with and without *H.pylori* infection (data not shown), suggesting that mucosal alterations in later stages of Correa's cascade might be too advanced and probably not anymore dependent on *H.pylori* status in gastric mucosa.

### MiRNA expression in different gastric regions in gastric cancer patients

Having shown an increased expression of miR-155 and miR-223 in the antrum and the corpus mucosa from patients with GC (Figure 2), we further evaluated the expression of these miRNAs in different locations within the stomach of GC patients (corpus, antrum, tumor surrounding non-tumor tissues (NT) and tumor tissues). Interestingly, the expression of miR-21 showed a different pattern compared to miR-155 and miR-223. While miR-21 expression increased from corpus to antrum to tumor tissue (p < 0.05 for NT vs. tumor in *posttest*), miR-155 and miR-223 showed the highest expression in NT compared to corpus and antrum, or even to the tumor itself (Suppl. Figure S3).

### Summary miRNA expression score

**Figure 4:**
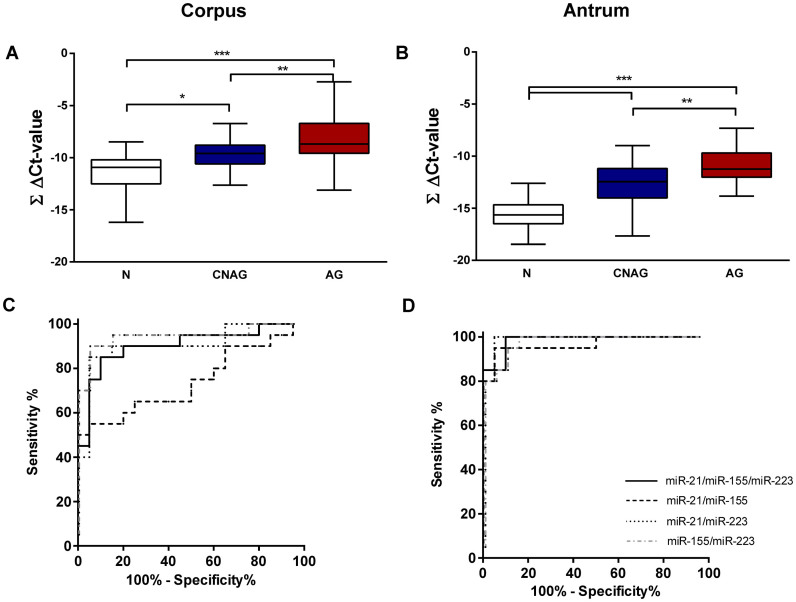
miRNA expression scores distinguish atrophic gastritis from normal mucosa. (A and B) ΔCt-values of miR-21, miR-155, and miR-223 were added together to calculate the ∑ ΔCt-value following normalization of each miRNA to RNU6b. Various summary scores have been used to calculate receiver operating characteristics for (C) corpus and (D) antrum. N – controls (n = 19); CNAG – chronic non-atrophic gastritis (n = 25); AG – atrophic gastritis (n = 20). ***-p < 0.0001; **-p < 0.001; *- p < 0.05.

Based on the observation that the studied miRNAs are increased in gastric preneoplastic precursor conditions, we questioned if a simple and easily applicable miRNA expression score might have diagnostic potential and therefore be useful for the risk stratification of patients with mucosal abnormalities. For this purpose, we combined ΔCt-values of the studied miRNAs into a single summary score for different combination of miRNAs with each other (ΔCtmiR-21 + ΔCtmiR-155 + ΔCtmiR-223), creating the pooled miRNA expression score (ΣΔCt-value, Figure 4A and B), which may facilitate the generation of individualized risk stratification. The ΣΔCt-score was significantly higher in CNAG and AG compared to N both in corpus (−11.37 ± 1.76 vs. −9.61 ± 1.47 vs. −8.12 ± 2.26, p < 0.0001; for N, CNAG and AG, respectively) and antrum (−15.69 ± 1.53 vs. −12.86 ± 2.39 vs. −10.97 ± 1.79; p < 0.0001). Translating this into the fold change, the mean score difference between AG and N is approximately 8-fold for corpus and 10-fold for antrum. To evaluate the diagnostic accuracy of this score, we performed a ROC-curve analyses for various combinations of the scores for corpus and antrum independently (ΔCtmiR-21 + ΔCtmiR-155 + ΔCtmiR-223; ΔCtmiR-21 + ΔCtmiR-155; ΔCtmiR-21 + ΔCtmiR-223 and ΔCtmiR-155 + ΔCtmiR-223). Especially the combination of all 3 miRNAs reached area under the curve (AUC) values of 0.9025 (95% CI 0.802–1.003) for the corpus, and 0.985 (95% CI 0.9577–1.012) for the antrum (Figure 4C and D). Among available tools, OLGA and OLGIM staging systems have been proposed for prediction of progression for gastric cancer development. To evaluate if the miRNA summary score may potentially be useful for prediction of GC development we compared its performance with the OLGA and OLGIM scores in our patients. We show that there is significant correlation between the extent of atrophic gastritis and intestinal metaplasia defined by OLGA and OLGIM scores, respectively, and the miRNA expression score in gastric mucosa (Supl. Figure 4A and B).

### Validation of differential miRNA expression in an independent cohort

**Figure 5:**
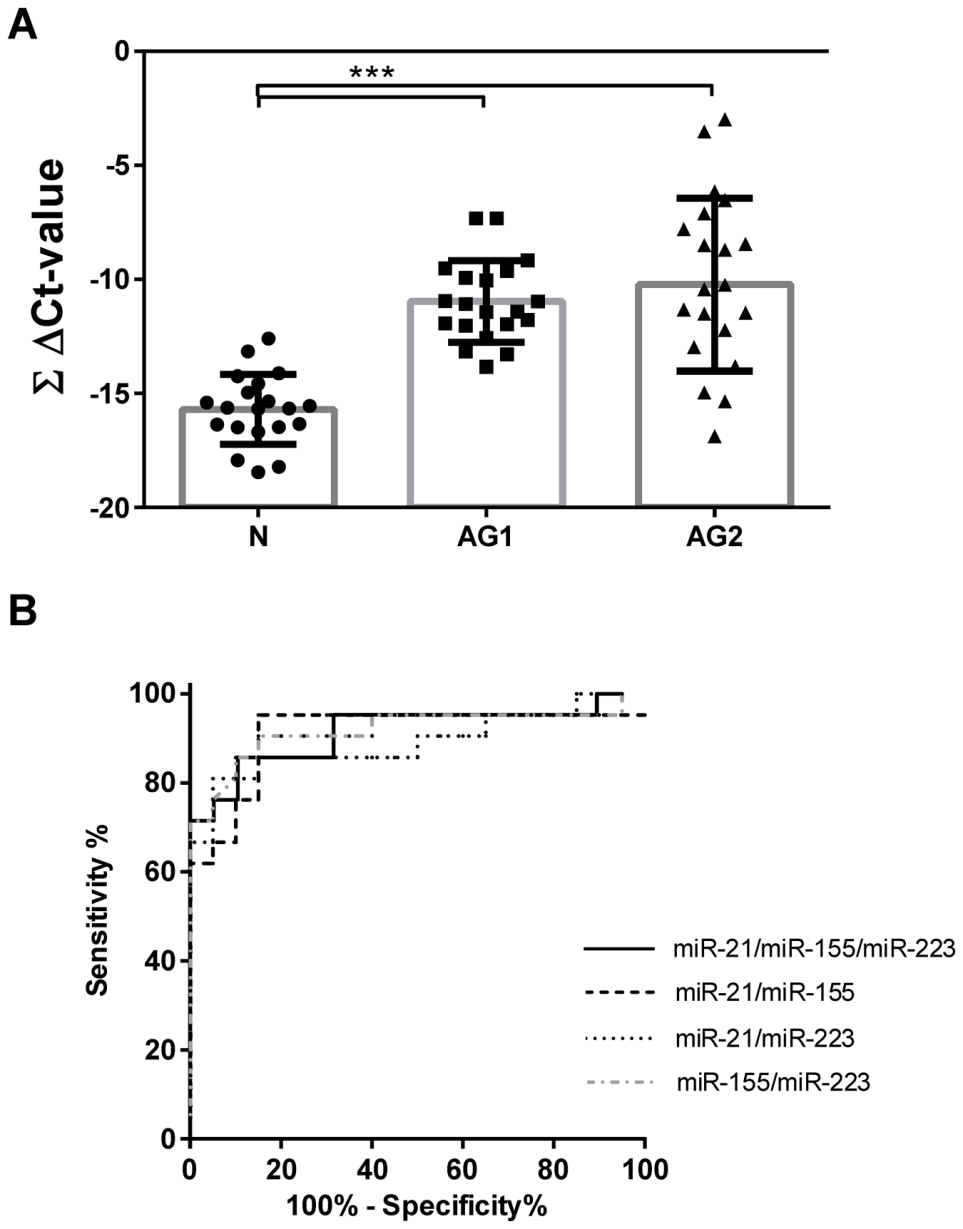
Validation analyses of differential miRNA expression and receiver operating characteristics. (A) ΔCt-values for each miR-21, miR-155, and miR-223 were summarized to calculate the ∑ ΔCt-values for normal cohort (N; n = 19), for patients with atrophic gastritis from the first cohort (AG1; n = 20) and for the validation cohort (AG2; n = 21) from the antrum mucosa. (B) Receiver operating characteristics were calculated for the second cohort of patients with histologically confirmed atrophic gastritis using various summary scores. ***-p < 0.0001.

To validate the results to diagnostic accuracy, we analyzed an independent set of 21 antrum biopsies from patients with AG. As shown on the Figure 5, antral biopsies from patients with AG from the first cohort (AG1) and the independent validation cohort (AG2) showed both significantly higher miRNA ΣΔCt-value compared to samples from patients with normal mucosa (AG1: −10.97 ± 1.79 and AG2: −10.23 ± 3.78 vs. N: −15.69 ± 1.53, p < 0.0001). No differences were observed between AG1 and AG2. In the Figure 5B the calculations are presented in similar way as in Figure 4C and D. In similar fashion, the ROC curve analyses evaluating the miRNA expression ΣΔCt-value revealed an AUC of 0.9148 (95% CI 0.82 ± 1.010) for the independent cohort (AG2), which was similar to the first cohort, further supporting the diagnostic potential of miRNA expression analyses.

## Discussion

In the present study we evaluated the expression of three miRNAs that are frequently deregulated in gastric cancer patients and are potentially related to chronic inflammation of the gastric mucosa. We showed that pronounced changes in the expression of miR-21, miR-155 and miR-223 occur in relation to a stepwise progression of *H.pylori* induced gastric preneoplastic conditions from CNAG to AG, if compared to normal non-affected mucosa. Alterations of the studied miRNAs may probably be related not only to a process of carcinogenesis, but could be part of the global mucosal process of the stomach, as these changes were also present in non-tumor mucosa from corpus and antrum of GC patients. In particular, upregulation of miR-155 and miR-223 was strongly related to *H.pylori* infection. As proof of principle, we demonstrated that the combination of these three miRNAs may be used as molecular biomarker for atrophic gastritis. Nevertheless, we also demonstrate that the gastric corpus and the antrum mucosa show distinct miRNA expression patterns. Therefore, careful collection, characterization and analysis of the gastric mucosa at different sites is essential.

The characterization of the gastric mucosa provides unique insight into the process of gastric carcinogenesis. The role of miRNAs in GC has been extensively studied in this regard^7^. However, only few studies were dedicated to miRNA expression in chronic gastritis and preneoplastic conditions. In currently available studies, miRNA expression has been analyzed in chronic gastritis (with unknown *H.pylori* status)^15^, in samples from *H.pylori*-infected antrum mucosa^18^, and in antrum samples of patients with duodenal ulcer as well as *H.pylori*-related dyspeptic conditions (subjects with intestinal metaplasia or atrophy where excluded from these analyses)^22^. Shiotani et al. focused specifically on high risk preneoplastic changes in subjects who previously underwent endoscopic resection of early gastric cancer^23^. In our study, we applied a systematic approach using biopsies from different sites of the stomach to characterize the expression of miRNAs in well-defined preneoplastic and neoplastic conditions in a prospective manner, which has not been performed in that form before.

For our analyses we selected three miRNAs related either to GC (miR-21, miR-223)^15^,^20^,^24^,^25^, *H.pylori* infection (miR-155, miR-223)^17^ or inflammation of the gastric mucosa^15^ (miR-155 and miR-223)^17^,^26^ based on previously published data. Furthermore, miR-223 has been identified earlier as a myeloid-specific miRNA with fine-tuner function during inflammatory processes^27^. In addition, increased miRNA expression of miR-155 has been shown following profiling analyses of chronic gastritis or gastric cancer tissues^15^. Based on that knowledge, several groups have further elaborated on the understanding of the functional role of miR-155. In particular, miR-155 has been shown to play an essential role in T-cell-mediated control of *H.pylori* infection^17^. Oertli et al. demonstrated that miR-155^−/−^ mice develop less severe immunopathology such as AG, epithelial hyperplasia and IM in response to *H.pylori* infection^17^. Data from our study are in strong correlation with these observations as we observed a stepwise increase of miR-155 expression from normal mucosa to CNAG and to AG in our prospective cohort, independent from the sampling site within the stomach. Interestingly, the expression in antrum and corpus mucosa from subjects with GC was slightly lower than observed in patients with AG. Similar changes were also found for miR-223, suggesting a crucial role of inflammation-involved miRNAs in the progress of Correa's cascade.

*H.pylori* infection is the key factor that leads to chronic gastritis and an increased risk of tumor development in the stomach^28^. Therefore, careful assessment of the association of miRNA expression with *H.pylori* status is needed. In concordance with previous observations^22^, we detected a strong association of both miR-155 and miR-223 expression with *H.pylori*-induced chronic gastritis, but not with *H.pylori*-negative gastritis. Based on our results, we suggest a specific *H.pylori*-related mechanism which may involve the gastric epithelium, inflammatory cells or both. Interestingly, miR-21 expression did not differ significantly between *H.pylori* positive and negative subjects. However, subjects with *H.pylori*-negative gastritis showed only marginal changes in miR-155 and miR-223 expression compared to normal, non-inflamed mucosa. Recent reports demonstrated a causal relationship between *H.pylori* and miRNA expression. It was shown that *H.pylori* CagA can induce aberrant epigenetic silencing of let-7 expression contributing to Ras upregulation and carcinogenesis^29^. To answer if and at what magnitude miR-155 and miR-223 may affect the gastric mucosa in response to *H.pylori* infection, further systematic and functional studies are needed. The influence of *H.pylori* strain-specific virulence factors need to be clarified.

MiR-21 is one of the most deregulated miRNAs in the process of carcinogenesis. This miRNA is frequently upregulated in gastric tumors^13^,^30^ and was detected as circulating miRNA in plasma^8^ or gastric fluids of GC patients^8^,^31^. We show here that miR-21 is upregulated in tumor tissues compared to normal mucosa of healthy subjects, or non-tumor mucosa of gastric cancer patients. However, these results might depend on the sampling location. In comparison to miR-223 and miR-155, expression changes of miR-21 were primarily found in the antrum and were highest in tumors. Whether this may be related to neoplasia-specific process needs to be further clarified.

Corpus predominant gastritis, AG and intestinal metaplasia are the gastric mucosal conditions that are associated with increased risk for gastric cancer development^32^. According current recommendations for the management of precancerous conditions of the stomach (MAPS), a follow-up endoscopy with a rigorous biopsy protocol is proposed for patients showing these conditions^6^. Taking into account the need of a lifelong endoscopic surveillance, the clinical utility and the cost of this approach remains a challenge in everyday practice and the search for non-invasive biomarkers for previously mentioned high risk conditions is still in progress. Currently available tools, such as evaluation of pepsinogen I/II levels are, with regional differences, not widely used in clinical practice because of the limited sensitivity and relatively high costs^33^, and alternative tools are urgently needed. Here, we performed a first exploratory step and evaluated mucosal miRNA expression as a diagnostic tool for AG. To overcome the limitation of single molecule analyses, we combined all three studied miRNAs creating a simple summary miRNA expression score. Based on these values the AUC was 0.9 for the corpus and 0.98 for the antrum, indeed suggesting potential diagnostic usefulness as further supported by the results from the independent cohort of patients with AG. In comparison to the globally used histological evaluation of gastric mucosa samples by a pathologist, the use of a miRNA-based mucosal biomarker for the detection of AG may be not sufficiently attractive. However, this approach may still be of high value as simple, objective automated systems can be established for mucosal miRNA characterization, while histological evaluation needs histological sample preparation in addition to experienced and trained pathologists. Whether this approach may become convincing/realistic and could contribute to the diagnosis or surveillance of the patients with preneoplastic conditions in high risk regions such as Asia or East Europe or globally, needs definitely further investigation. We provide in this study a proof-of-principle observation with a relatively small sample size and further studies need to compare the results with existing serological markers (pepsinogen I/II) and histological parameters (updated Sydney classification, OLGA and OLGIM staging systems). Furthermore, additional studies are needed to evaluate if miRNA changes in the gastric mucosa correlate with systemic changes and if it could be used for development of non-invasive diagnostic tests. Besides, longitudinal studies are needed to establish a prognostic role of this marker panel for high risk gastritis subjects, for example to predict the reversal of the mucosal alterations after *H.pylori* eradication or progression to GC. In addition, with exception of one patient, all of the patients with AG had intestinal metaplasia. It would be further important to dissect this condition specifically, as it is frequently considered as “point of no return”. In this proof-of-principle study, we focused on the expression of three selected miRNAs and additional studies are urgently needed to gain the global overview of miRNA expression changes and related epigenetic regulation such as DNA methylation^7^.

As we mentioned earlier, most of the currently available studies focused either on antrum mucosa or the region of sampling was not specified. Surprisingly, we found significant difference in the miRNA expression in the normal mucosa of healthy subjects. All of the investigated miRNAs were significantly higher expressed in the corpus compared to the antrum in both subjects with normal mucosa or chronic gastritis. These data strongly suggest that the region of sampling within the stomach needs to be documented carefully taking into account these local regional differences found in our study.

In conclusion, we have shown that miR-21, miR-155 and miR-223 are differentially expressed in preneoplastic and neoplastic gastric mucosa. A gradual increase of the miRNA expression from normal to atrophic mucosa correlates with mucosal stages of Correa's cascade suggesting miRNAs as potential diagnostic/prognostic biomarkers. Close association between *H.pylori* infection and miR-155/miR-223 suggests a functional role of the specific *H.pylori*-mediated inflammatory environment. However, differences in the miRNA expression pattern between corpus and antrum should be taken into account in future studies as revealed by systematic mucosal comparison.

## Methods

### Ethics

This prospective study was performed according to the “World Medical association Declaration of Helsinki – Ethical Principles for Medical Research Involving Human Subjects”. The study protocol was approved by the Institutional Review Board of Otto-von-Guericke University Magdeburg (Number 80/11). Every participant gave his written informed consent prior to study inclusion.

### Study design

Patients undergoing upper gastrointestinal endoscopy at the Department of Gastroenterology, Hepatology and Infectious Diseases at the Otto-von-Guericke University Magdeburg, Germany, were asked to participate in the study. Subjects with the following criteria were excluded: other cancers than GC, preoperated stomach, acute bleeding, former radiation therapy of the upper abdomen, oral anticoagulation, immunosuppressive or antibiotic therapy. Samples were collected between July 2011 and September 2013. From the total study population which consisted in October 2012 of 257 patients, random high-quality samples from a total of 80 patients were selected for this proof-of-principle study. Patients with CNAG (±H.*pylori*, n = 25), AG (±IM, n = 20), and GC (±IM, n = 16) and 19 controls (N) were included in this analysis. All subjects were Caucasians of European origin. To validate the differences in miRNA expression with the focus on biomarker potential, a second independent cohort of 21 patients with AG were included (Suppl. Figure S1). Healthy controls were defined as normal mucosa without any active inflammation or moderate/severe chronic gastritis according to the updated Sydney classification, and were negative for *H.pylori* infection in all applied tests. Clinical and demographical data are shown in Table 1.

### Endoscopy and histological evaluation of samples

Gastric biopsies from consecutive patients were obtained in routinely performed gastroscopies. The typing and grading of gastritis including intestinal metaplasia (IM) was performed according to the updated Sydney classification^34^. GC patients were defined by the International Classification of Diseases for Oncology and Lauren criteria. The biopsies (from antrum and corpus) were immediately snap-frozen in liquid nitrogen and stored at −80°C until usage for molecular analysis. Another sample per localization was formalin-fixed and further processed for routine histopathological assessment. In GC patients, if possible, an additional set of biopsies including tumor and adjacent non-tumor mucosa surrounding the tumor (NT) were collected as snap-frozen for molecular analysis or formalin-fixed for histopathological assessment.

### H.pylori diagnosis

Status of *H.pylori* infection was determined by rapid urease test, serology, histology and microbiology. Patients were defined as *H*.*pylori* positive with positive microbiology and/or positive histology and/or positive serology. Patients with no direct detection of *H.pylori* in microbiology or histology and negative serology were defined as *H.pylori* negative. For *H.pylori* culture gastric specimens were stored in 0.9 vol% isotonic sodium chloride solution (Berlin-Chemie AG, Berlin, Germany). Biopsies were cultured on Columbia-agar-based medium with and without antibiotic supplement (10 mg/ml vancomycin, 1 mg/ml nystatin, and 5 mg/ml trimethropin). Plates were incubated for a maximum of 10 days under microaerophilic conditions (37°C, 5% CO_2_) and bacterial growth was checked every 2–3 days. *H.pylori* was identified by typical morphology in gram-negative staining and positive urease, oxidase, and catalase test.

### RNA isolation and miRNA quantification

Total RNA (including miRNA) was isolated from gastric biopsies using the Qiagen RNeasyPlus Universal Mini Kit (Qiagen, Hilden, Germany) according to the manufacturers' instructions. Briefly, frozen biopsy samples were homogenized in QIAzolLysis Reagent (Qiagen, Hilden, Germany) using the TissueRuptor and total RNA was precipitated using Chloroform and aqueous phase was mixed with 1.5 volumes of 100% Ethanol. RNA quality was evaluated using UV-spectrophotometry. Samples were stored at −80°C until further analysis. Quantification of miRNA expression was assessed using TaqMan miRNA assay (Applied Biosystems, CA, USA) or SYBRgreen methods as described previously (34). According to the manufacturer's protocol, approximately 20 ng of total RNA were reverse transcribed and quantitative real-time PCR analyses were completed using BioRad CFX Cycler System (BioRad, CA, USA). Reverse Transcription was done according to the manufacturer's protocol. Following primers were used for the analyses: miR-21 (21); miR-155 (TaqMan Assay ID: 002623); miR-223 (TaqMan Assay ID: 002295). MiRNA expression levels were further normalized to small nuclear RNA RNU6b^35^.

### Statistical Analysis

Data analysis was performed using GraphPad Prism 6.0 software (San Diego, CA, USA). Data are reported as mean ± SD. The Mann-Whitney U-test and the Kruskal-Wallis analyses of variance were used to analyze the statistical significance for unpaired group or multiple group comparison, respectively. Wilcoxon's test was used for analyses of paired groups. Appropriate Dunn's multiple comparison test was used for the post hoc analyses. ANOVA analyses of variance were used for comparison of normally distributed data. Receiver operating characteristic (ROC) analyses were performed to evaluate the diagnostic performance of the miRNA expression pattern in gastric biopsies defined by the Area under the curve (AUC). A two-sided p-value <0.05 was regarded as significant.

## Author Contributions

A.L., T.W., P.M. study concept and design, analyses and interpretation of data; A.L., W.S., P.M. drafting of manuscript and obtaining funding; M.V., J.B. provided clinical materials; A.L., C.L., W.S. performed the experiments; all authors approved the final version of the manuscript.

## Supplementary Material

Supplementary Information Supplementary Information

## Figures and Tables

**Table 1:** Clinical characteristics of patients for microRNA expression analyses

	Total	N	CNAG	AG	GC
	n (%)	n (%)	n (%)	n (%)	n (%)
**Patients**	80 (100%)	19 (23.75%)	25 (31.25%)	20 (25%)	16 (20%)
**Sex**					
Women	48	12	17	14	5
Men	32	7	8	6	11
**Age (mean)**	58.6	50.5	54.2	63.8	68.6
***H.pylori***					
positive	41	0	21	12	8
negative	39	19	4	8	8
**Intestinal metaplasia**	27	0	0	19	8
**Lauren**'**s classification**					
Diffuse		-	-	-	5
Intestinal		-	-	-	9
other		-	-	-	2
**Tumor localization/Region**					
Cardia		-	-	-	7
Corpus		-	-	-	6
Antrum		-	-	-	3

N: patients with normal mucosa; CNAG: chronic non-atrophic gastritis; AG: atrophic gastritis; GC: gastric cancer.
