# Supplementary material for: Differential expression of microRNAs in preneoplastic gastric mucosa
Source: Sci Rep. 2015 Feb 5;5:8270. doi: 10.1038/srep08270 (PMC4317705; doi:10.1038/srep08270)

Supplementary Information

Differential expression of microRNAs in preneoplastic gastric mucosa

Alexander Link*, Wiebke Schirrmeister, Cosima Langner, Mariya Varbanova, Jan Bornschein, Thomas Wex, Peter Malfertheiner

Department of Gastroenterology, Hepatology and Infectious Diseases, Otto-von-Guericke University, Magdeburg, Germany

Correspondence:

Dr. Alexander Link

Department of Gastroenterology, Hepatology and Infectious Diseases

Otto-von-Guericke University

Leipziger Str. 44,

39120 Magdeburg (Germany)

Phone: +49-391-6713106

Fax: +49-391-6713105

Email: alinkmail@gmail.com

Figure legends

Supl. Figure S1. Study design. In discovery phase, we first performed systematic characterization of miRNA expression in various cohorts of patients. In the training phase, we calculated various miRNA expression scores to estimate potential diagnostic usefulness to discriminate atrophic gastritis from normal controls. In the validation phase, we performed conformational analyses to validate the results using independent cohort of antrum biopsies from patients with atrophic gastritis.

Supl. Figure S2. MiRNA expression in the normal mucosa and gastric cancer. Expression values of (A) miR-21, (B) miR-155 and (C) miR-223 are shown as normalized $2^{\Delta dCt}$-values. RNU6b was used for normalization. ***-p<0.0001; **-p<0.001; *- p<0.05. N – controls (n=19) from both corpus and antrum; GC – gastric cancer tissue (n=10).

Supl. Figure S3. MiRNA expression in different regions of the stomach from gastric cancer patients. The expression of miR-21 (A), miR-155 (B) and miR-223 (C) was evaluated in corpus (n=14), antrum (n=16), near tumor (NT; n=7) or in tumor tissues (n=10) from GC patients. The data are presented as $2^{\Delta dCt}$-values normalized to RNU6b. ***-p<0.0001; **-p<0.001; *- p<0.05.

Supl. Figure 4. Correlation between miRNA expression scores and OLGA and OLGIM grading scores in gastric mucosa. (A and B) ΔCt-values of miR-21, miR-155, and miR-223 were added together to calculate the ∑ ΔCt-value following normalization of each miRNA to RNU6b. Correlation analyses between miRNA expression score and (A) OLGA and (B) OLGIM grading scores were performed using Spearman’s test.

Suppl. Figure S1.

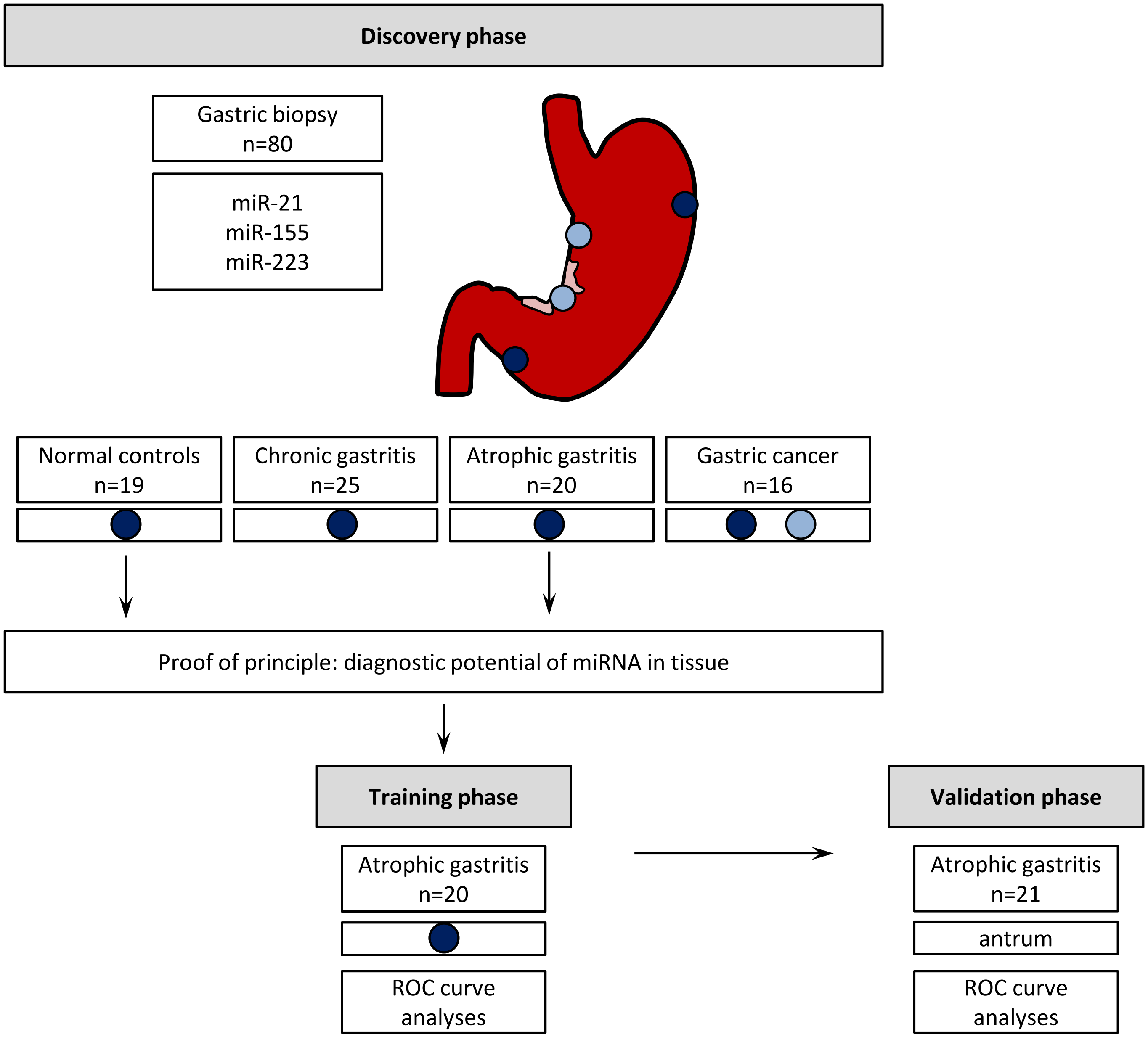

Suppl. Figure S2.

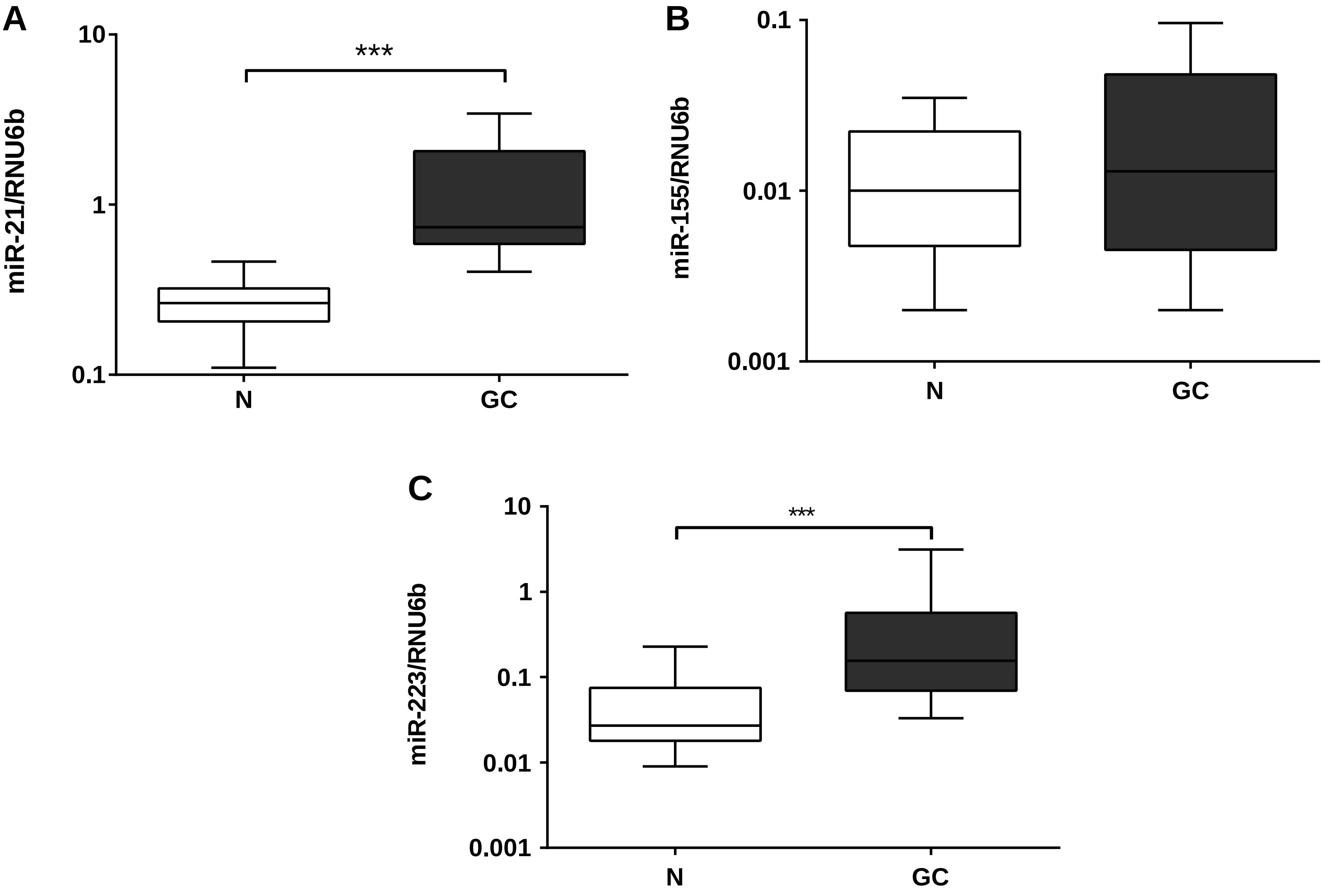

Suppl. Figure S3.

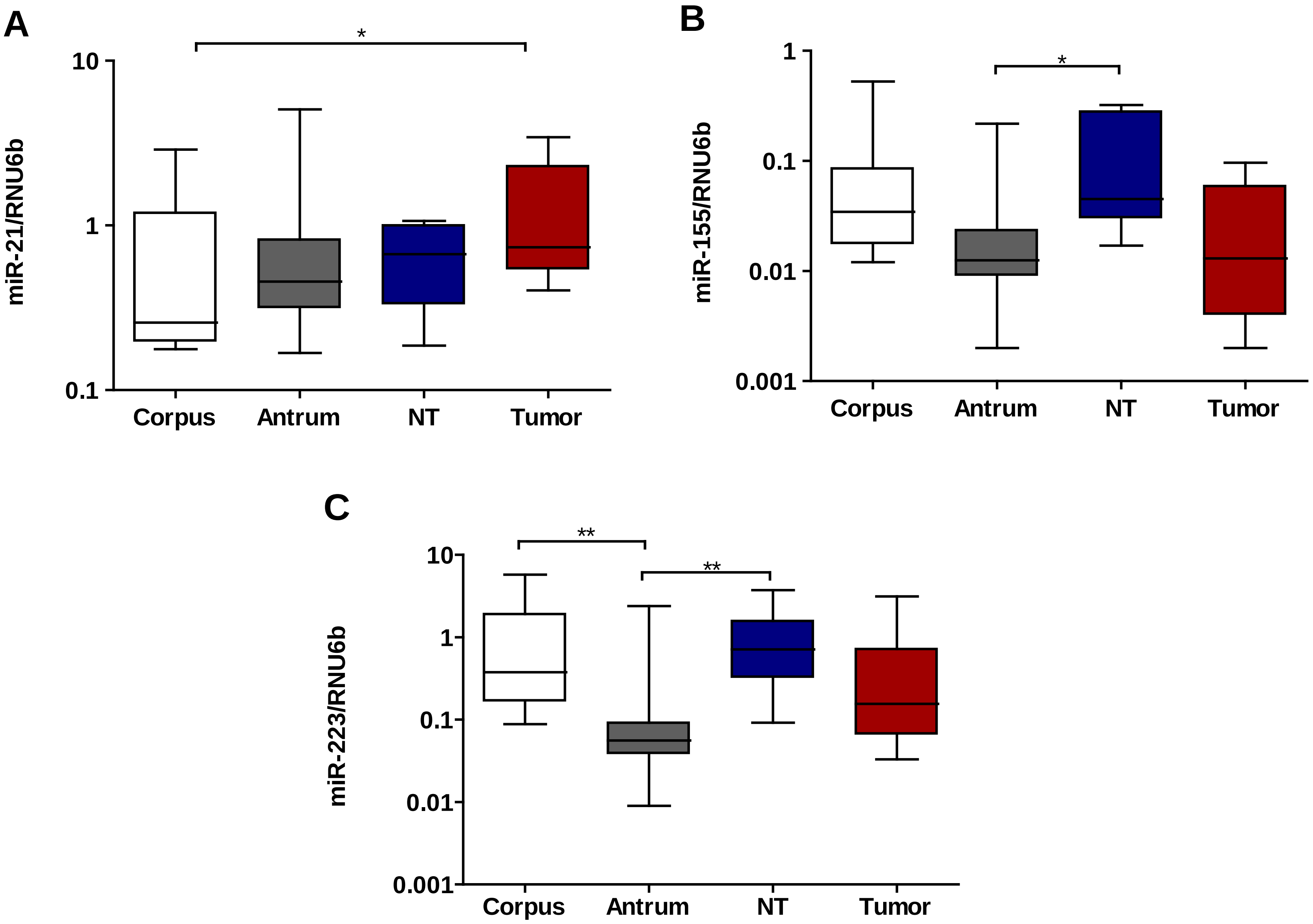

Suppl. Figure S4.

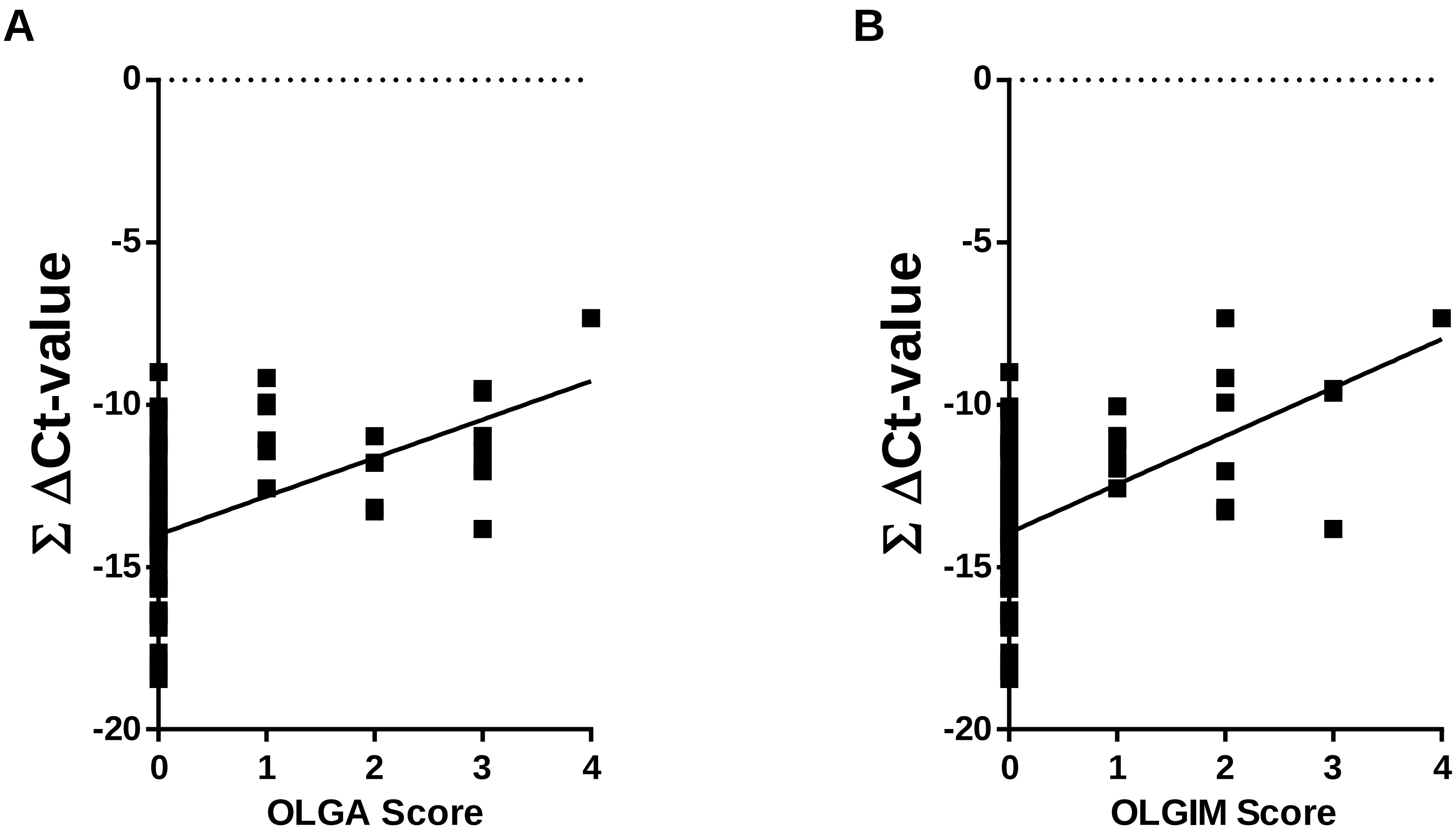

Supplement: Supplementary Information [file srep08270-s1.pdf]
